# Limited genetic and antigenic diversity within parasite isolates used in a live vaccine against *Theileria parva*

**DOI:** 10.1016/j.ijpara.2016.02.007

**Published:** 2016-07

**Authors:** Johanneke D. Hemmink, William Weir, Niall D. MacHugh, Simon P. Graham, Ekta Patel, Edith Paxton, Brian Shiels, Philip G. Toye, W. Ivan Morrison, Roger Pelle

**Affiliations:** a The Roslin Institute, Royal (Dick) School of Veterinary Studies, University of Edinburgh, Easter Bush, Roslin, Midlothian EH25 9RG, UK; b College of Medical, Veterinary and Life Sciences, Institute of Biodiversity Animal Health and Comparative Medicine, University of Glasgow, Henry Wellcome Building, Garscube Campus, Bearsden Road, Glasgow G61 1QH, UK; c The International Livestock Research Institute, PO Box 30709, Nairobi, Kenya

**Keywords:** *Theileria parva*, Vaccination, Cattle, Antigenic diversity, Gene sequencing, Satellite DNA

## Abstract

An infection and treatment protocol is used to vaccinate cattle against *Theileria parva* infection. Due to incomplete cross-protection between different parasite isolates, a mixture of three isolates, termed the Muguga cocktail, is used for vaccination. While vaccination of cattle in some regions provides high levels of protection, some animals are not protected against challenge with buffalo-derived *T. parva*. Knowledge of the genetic composition of the Muguga cocktail vaccine is required to understand how vaccination is able to protect against field challenge and to identify the potential limitations of the vaccine. The aim of the current study was to determine the extent of genetic and antigenic diversity within the parasite isolates that constitute the Muguga cocktail. High throughput multi-locus sequencing of antigen-encoding loci was performed in parallel with typing using a panel of micro- and mini-satellite loci. The former focused on genes encoding CD8^+^ T cell antigens, believed to be relevant to protective immunity. The results demonstrate that each of the three component stocks of the cocktail contains limited parasite genotypic diversity, with single alleles detected at many gene/satellite loci and, moreover, that two of the components show a very high level of similarity. Thus, the vaccine incorporates very little of the genetic and antigenic diversity observed in field populations of *T. parva*. The presence of alleles at low frequency (<10%) within vaccine component populations also points to the possibility of variability in the content of vaccine doses and the potential for loss of allelic diversity during tick passage. The results demonstrate that there is scope to modify the content of the vaccine in order to enhance its diversity and thus its potential for providing broad protection. The ability to accurately quantify genetic diversity in vaccine component stocks will facilitate improved quality control procedures designed to ensure the long-term efficacy of the vaccine.

## Highlights

• Genotyping and gene sequencing reveal limited diversity in a live *Theileria parva* vaccine.
• Two of the vaccine components show a high level of similarity at all loci.
• The vaccine contains very little of the diversity found in field *T. parva.*
• The vaccine is suboptimal for generating immunity again diverse parasite strains.
• The presence of minor genotypic components poses difficulties for quality control.

## Graphical abstract

**Figure:**
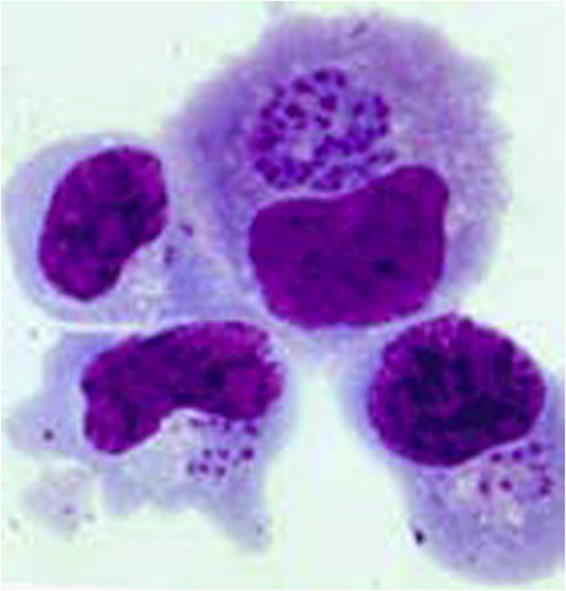

## 1 Introduction

Live vaccines are available for a number of economically important diseases of farm animals caused by protozoal parasites including *Babesia bovis*, *Theileria parva* and *Theileria annulata* in cattle, *Toxoplasma gondii* in sheep and *Eimeria* spp. in poultry (reviewed by McAllister, 2014). Extensive efforts to develop alternative, more easily manufactured, vaccines based on use of defined antigens, have met with limited success. Hence, vaccination against these diseases will continue to rely on the available live vaccines for the foreseeable future. Similar slow progress in developing subunit vaccines for malaria has also prompted recent efforts to develop a live vaccine using *Plasmodium* sporozoites harvested from infected mosquitos (Hoffman, 2012, Hodgson et al., 2015). Antigenic heterogeneity is an important feature of some of these protozoa, notably *T. parva* (Morrison et al., 2015) and *Eimeria tenella* (Blake et al., 2015)*,* requiring the inclusion of more than one parasite genotype in the vaccines.

*Theileria parva,* transmitted by *Rhipicephalus appendiculatus* ticks, causes an acute lymphoproliferative disease of cattle, which is a major constraint to livestock production throughout a large part of east and southern Africa. Due to the fatal nature of the disease in cattle and shortcomings of therapeutic drugs and measures to control tick infestation, vaccination offers the most sustainable means of controlling the disease. A method of vaccination, involving infection of cattle with *T. parva* sporozoites and simultaneous treatment with long-acting tetracycline, referred to as the infection and treatment method, was developed over 40 years ago (Radley et al., 1975). The initial work demonstrated that immunisation with one parasite isolate resulted in solid, long-lasting immunity against challenge with the same isolate; however, a proportion of animals remained susceptible to challenge with other isolates (Radley et al., 1975). Following a series of immunisation and challenge experiments, a combination of three parasite isolates was identified, which generated immunity against experimental challenge with a range of *T. parva* isolates of cattle origin (Radley et al., 1975) and against field challenge (Radley, 1978). This vaccine, commonly referred to as the Muguga cocktail, contains the Muguga, Kiambu 5 and Serengeti isolates of *T. parva*. The former two isolates were derived by feeding ticks on infected cattle, whereas the Serengeti component was originally isolated by feeding ticks on an African buffalo after which it was adapted to cattle through tick passage (Young and Purnell, 1973). Until recently, use of the Muguga cocktail vaccine in the field has been limited, largely due to the complex process involved in production and quality control of sporozoites and the requirement for a cold chain to store and distribute the vaccine. However, a recent initiative to establish a centre for vaccine production (http://www.galvmed.org/en/livestock-and-diseases/livestock-diseases/east-co) and systems to facilitate its distribution have led to increased field uptake.

Despite evidence of efficacy of the Muguga cocktail vaccine in some field settings (Di Giulio et al., 2008, Martins et al., 2010), failure to protect a proportion of immunised animals has been observed following exposure to tick challenge in a paddock experimentally seeded with buffalo-derived *T. parva* (Radley et al., 1979). Moreover, in more recent studies, vaccinated animals exposed to natural field challenge in sites grazed by buffalo were found to be susceptible to challenge (Bishop et al., 2015, Sitt et al., 2015). As indicated previously (Bishop et al., 2001), more detailed knowledge of the genetic composition of the Muguga cocktail vaccine is required, not only to understand how the vaccine content relates to its ability to protect against field challenge with *T. parva* but also to facilitate quality control during vaccine production. Although the protective capacity of the vaccine is believed to reflect genotypic diversity of the constituent parasites at antigen-encoding loci, information on the genetic composition of the parasite populations within each of the vaccine components is limited. Allelic polymorphism of three genes encoding antigens recognised by immune sera has been documented in the components of the Muguga cocktail using restriction fragment length polymorphism analysis and/or monoclonal antibody reactivity (Geysen et al., 1999, Bishop et al., 2001). More recently, a whole-genome sequencing approach has been applied to characterising Muguga cocktail component stocks (Norling et al., 2015). While this confirmed genetic diversity among the components, intra-component diversity was not detected beyond the expected error level of the sequencing platform employed. The most discriminant method for examining intra-component genetic diversity to date has been the use of micro- and mini-satellite markers; these have been employed to demonstrate diversity both between and within the vaccine cocktail components (Oura et al., 2004, Oura et al., 2007, Patel et al., 2011).

The short ‘variable number of tandem repeat’ regions detected by micro- and mini-satellite genotyping are broadly distributed in eukaryotic genomes (Tautz and Renz, 1984). As they possess a relatively high rate of mutation, they represent useful markers for high-resolution molecular fingerprinting of eukaryotic pathogens. These loci, for the most part, do not encode proteins involved in stimulating host immunity and can only be used as a proxy for assessing antigen diversity if they are closely physically linked to antigen-encoding loci. Moreover, micro- and mini-satellite genotyping is not well suited to detecting low abundance genotypes within mixed parasite populations, since minor alleles can be difficult to differentiate from PCR artefacts.

Previous studies provided evidence that CD8^+^ T cell responses specific for parasitised lymphoblasts are key mediators of immunity generated by infection and treatment vaccination (McKeever et al., 1994, Taracha et al., 1995a). Recently, a number of *T. parva* antigens recognised by CD8^+^ T cells from immune animals were identified and several of these antigens have been shown to be polymorphic in field populations (Pelle et al., 2011). In this study, high-throughput multi-locus sequencing of antigen-encoding loci was performed, focusing on genes encoding parasite antigens believed to be relevant to protective immunity. A panel of micro- and mini-satellite loci was also utilised in order to gain a broad understanding of genetic diversity within the Muguga cocktail.

## 2 Materials and methods

### 2.1 Samples and DNA extraction

Four batches of Muguga cocktail vaccine stabilate (ILRI 0801–ILRI 0804) and the corresponding reference stabilates (Muguga – 4230; Serengeti – 4229; Kiambu 5 – 4228), prepared previously as described by Patel et al. (2016), were used in this study. Both the reference stabilates and the vaccine stabilates had been prepared from ticks infected with the same parent stabilates (Muguga – 73, Serengeti – 69 and Kiambu 5 – 68). Three batches of infected ticks, each obtained from cattle infected with one of the component stabilates (68, 69 and 73) were used at different times to prepare the reference and vaccine stabilates. The latter were prepared from a mixture of ticks from the three batches. Following moulting to adults, ticks from each of the batches were fed on rabbits for 4 days and a sample of the ticks was used to determine the infection rates in each batch of ticks, based on examination of dissected salivary glands. The final mixture of the ticks was prepared to ensure there were equivalent numbers of acini infected with parasites from each of the component stabilates. The tick mixture was homogenised, suspended in 7.5% glycerol and cryopreserved in aliquots in liquid nitrogen (Cunningham et al., 1973). The four vaccine batches were prepared from a subset of the same batches of ticks processed 1 week apart. Following preparation of the vaccine stabilates, the three reference stabilates were prepared separately from ticks infected with each of the three parasites. These stabilates (4228, 4229 and 4230) are currently being used to produce future batches of vaccine.

An additional sporozoite stabilate (3014) of the Marikebuni *T. parva* stock was also examined. This stock, which has been used on a more limited scale for vaccination by infection and treatment (Mutugi et al., 1989), was originally isolated from a field tick collection in the coastal district of Kenya and had undergone three laboratory passages through ticks (Irvin et al., 1983, Mutugi et al., 1989). This stock was used as a comparator in order to investigate how the diversity in a single isolate used for vaccination compares with that of the trivalent Muguga cocktail.

For DNA extraction, excess tick debris was first removed from thawed sporozoite stabilates by centrifuging at 100*g* for 5 min and the supernatant containing the sporozoites transferred to a clean Eppendorf tube. The sporozoites were pelleted at 16,000*g* before DNA was extracted using the Qiagen DNeasy Blood and Tissue Kit.

### 2.2 Micro- and mini-satellite DNA typing

PCR amplification was performed using micro- and mini-satellite markers described by Oura et al., 2003, Oura et al., 2005. Fifteen satellite markers distributed across the four chromosomes of the parasite genome were used, namely ms2, MS3, ms7, MS7, MS8, MS14, MS15, MS19, MS21, MS25, MS27, MS30, MS33, MS39 and MS40 (genome references provided in Katzer et al., 2010). PCR conditions were similar to those described previously, except that one primer of each primer pair was labelled at the 5′ end with the fluorophore FAM (Eurofins Genomics, UK). Allele sizes were determined using capillary flow electrophoresis and detection of the fluorophore signal from the labelled primer in each amplicon. This was done using Genescan™ technology (Applied BioSystems, UK) with the GSLIZ500 size standards. Only peaks in the range of 100–500 bp were considered for further analysis. The relative abundance of individual peaks was determined and expressed as a percentage of the total area under the curve for all peaks of the appropriate colour in the trace combined. Peaks with an area of less than 3% of the total area under the curve for all peaks were excluded. Traces were inspected and alleles were manually ‘called’.

*F*-statistic values were calculated from the mini- and microsatellite typing data from the different parasite stocks and were utilised to carry out principal co-ordinate analysis (PCoA). The Microsoft plug-in Genalex6 (Peakall and Smouse, 2012) was used to perform PCoA.

### 2.3 *Theileria parva* CD8^+^ T cell antigens

Ten *T. parva* genes (Tp1–Tp10) known to encode antigens recognised by immune CD8^+^ T cells were investigated. Identification of Tp1 (TP03_0849), Tp2 (TP01_0056), Tp4 (TP03_0210), Tp5 (TP02_0767), Tp7 (TP02_0244) and Tp8 (TP02_0244) antigens and their T cell epitopes has been reported previously (Graham et al., 2006, Graham et al., 2008). The same authors also identified a further two antigens, Tp3 and Tp6, corresponding to *T. parva* genome IDs TP01_0868 and TP01_0188, respectively. Tp9 and Tp10 (genome IDs TP02_0895 and TP04_0772, respectively) were identified subsequently by two of the current authors (ND MacHugh and SP Graham) using parasite-specific CD8^+^ T cells to screen a parasite cDNA library as described previously (Graham et al., 2006); Tp9 and Tp10 were shown to be presented by the 1^∗^02301 and 3^∗^00201 class I major histocompatibility complex (MHC) alleles, respectively, and a single CD8^+^ T cell epitope (Tp9_67–75_ – AKFPGMKKS; Tp10_419–427_ – NNPELIPVL) was identified in each by screening synthetic peptides as described elsewhere (Graham et al., 2008).

#### 2.3.1 Sanger sequencing

The full-length open reading frame (ORF) of the Tp2 and Tp9 genes, and a segment of the Tp1 gene, located between nucleotides 523 and 954, were sequenced from a series of cloned parasitised cell lines derived from each of the three constituent Muguga cocktail parasite stocks (Patel et al., 2011). The primers and PCR conditions used for amplification of the Tp1 and Tp2 sequences have been described previously (Pelle et al., 2011). Primers for Tp9 (forward: 5′-ATGAATGTTCTAACTACTGG-3′; reverse: 5′-TTATTGTTTTGTCCATGGTTTATTACG-3′) were designed based on the first 20 and the last 27 nucleotides of the Tp9 ORF from the Muguga reference isolate F100 TpM (GenBank accession number XM_760370). A second set of primers (forward: 5′-ACCCCATCCATTTGACAG-3′ and reverse 5′-CCTGGCATTAAGGTACTC-3′), designed to amplify a product of 369 bp of the genomic sequence of Tp9, with the forward primer located 78 bp downstream of the start codon, was used to generate PCR products from the Marikebuni stock (stabilate 3014). The PCR conditions used for Tp9 were as described for Tp1 and Tp2 (Pelle et al., 2011), except that annealing was performed at 55 °C for 1 min and the extension at 72 °C was for 90 s. The amplicons were processed and sequenced either directly or after cloning into a pGEM-T Easy plasmid vector (Promega) and sequenced as previously described (Pelle et al., 2011).

#### 2.3.2 Processing of samples for high throughput sequencing

A multi-locus sequence typing system was established using Roche (USA) 454 amplicon sequencing technology in order to identify allelic variants present at a low frequency in the vaccine stocks. Primers were designed to amplify segments of 10 genes (292–492 bp) known to encode antigens capable of recognition by CD8^+^ T cells (Tp1–Tp10). Regions containing known CD8 T cell epitopes were selected. The primers were tested for their ability to generate PCR products from a panel of 32 genotypically diverse cloned *T. parva*-infected cell lines. These clones were derived by limiting dilution from established *T. parva*-infected cell lines and included genetically distinct clones of the Marikebuni stock produced by Katzer et al. (2006) (*n* = 10), and clones derived during the course of the current study from isolates from cattle grazed in proximity of buffalo (*n* = 8) (Bishop et al., 2015) and isolates obtained from buffalo (*n* = 14) (Conrad et al., 1987). Specificity was tested using DNA from other *Theileria* spp., *T. annulata, Theileria buffeli, Theileria taurotragi* and *Theileria* sp. (buffalo), uninfected bovine peripheral blood mononuclear cells (PBMC) and uninfected *R. appendiculatus* ticks. Several primer combinations were tested and for nine genes, primer pairs were identified that gave a product of the expected size from each parasite clone which was specific for *T. parva* (see Table 1). In order to take account of genetic polymorphism at the locus encoding Tp2, two pairs of primers were designed to ensure amplification of all alleles. The same nucleotide positions were targeted by each primer pair in separate reactions and equal quantities of the two products were pooled prior to sequencing. A longer amplicon (562 bp) was obtained for Tp2 than for the other genes. Efforts to identify primers that would generate a suitably sized PCR product for Tp9 from all parasite clones were unsuccessful and therefore this gene was not pursued further.

Each primer was then modified to produce a fusion primer comprising three components, an adaptor sequence required for processing of the amplicon for sequencing, a 10 nucleotide sequence known as the Multiplex Identifier (MID) and the gene-specific primer sequence. The sequences of the adaptors and MIDs used were those described in the Roche Technical bulletin (http://454.com/downloads/my454). These fusion primers were then re-tested to confirm they yielded PCR products of the expected size.

Amplicons were generated for the nine genes (Tp1–Tp8 and Tp10) using the fusion primers in combination with a polymerase with proofreading capacity. The primer concentrations and annealing temperatures used in each of the PCR assays are shown in Table 1. For Tp1, Tp3, Tp4, Tp5, Tp6, Tp7 and Tp8, the reactions comprised 5 μl of 10× Fast start High Fidelity reaction buffer containing 18 mM MgCl_2_ (Roche) and 2.5 units of Fast start high fidelity enzyme blend (Roche), 1 μl of DNA and nuclease-free water in a final volume of 50 μl. The cycling conditions were as follows: 95 °C for 3 min followed by 30 cycles for 30 s at 95 °C, 30 s at the temperatures specified in Table 1 and 30 s at 72 °C, followed by a final extension at 72 °C for 7 min. The melting temperature for the primers designed for the amplification of Tp2 and Tp10 was outside the working range of the Fast start high fidelity enzyme blend (49 °C). Therefore products for these genes were obtained with a mixture of 1:1 Taq polymerase (Bioline, UK) and *Pfu* DNA polymerase (Promega, UK). The PCR comprised 10–20 pmol of each primer (see Table 1), 5 μl of custom PCR mix (Bioline), 1 μl of DNA and nuclease-free water in a final volume of 50 μl. The cycling conditions were as follows: 95 °C for 3 min followed by 35 cycles of 95 °C for 1 min, 49 °C for 1 min and 72 °C for 2 min, followed by a final extension at 72 °C for 7 min. PCR products were visualised under UV light in 1.5% agarose gels containing SafeView nucleic acid stain (NBS, Biologicals Ltd, UK) and bands corresponding to the product of interest were excised. The PCR products were then purified using the Promega Wizard Gel and PCR Clean-up System (Promega Corporation, USA). The purity of PCR products was assessed using Agilent DNA1000 chips on a 2100 Bioanalyser (Agilent Technologies, USA). PCR products were quantified using a picogreen assay (Thermo Fischer Scientific, USA). Equimolar quantities of PCR products were pooled and submitted to the Centre for Genomics Research (CGR), University of Liverpool, UK for Roche 454 sequencing.

### 2.4 Analysis of high throughput sequence data

Raw 454 sequencing data were processed using a custom bioinformatics pipeline. Briefly, reads for individual PCRs were extracted from multiplexed sequence datasets on the basis of the MID tags and primer sequences present within each read. As sequence quality was reduced towards the end of the relatively long Tp2 reads, and forward and reverse reads could not be paired with confidence, only the first 290 bases of the forward reads were analysed for this gene. The raw reads for a single gene in a single sample were then subjected to noise reduction (shhh.flows and shhh.seqs) and chimaera detection algorithms implemented on the Mothur platform (Schloss et al., 2009). Three different algorithms were used in order to maximise the chance of chimaera detection, namely Perseus, Chimera slayer and Uchime (Edgar et al., 2011, Haas et al., 2011, Quince et al., 2011). ‘Variants’ at a frequency below 0.4% or below five reads were removed from the dataset. These cut-offs were chosen based on control experiments where sequences were obtained from every gene following amplification from a clonal parasite stock. Resulting sequences were aligned using MUSCLE (Edgar, 2004) and visualised using the MEGA 5 phylogenetics suite (Tamura et al., 2011). Due to the presence of homopolymeric-length errors, which are intrinsic to 454 technology, single nucleotides were added to or deleted from sequence reads as necessary when the length of a homopolymer tract differed from that of the most abundant allele(s); not correcting such errors would have led to frame shifts and thus a change to the translated sequence and/or premature stop codons. Where single base pair insertions and deletions altered the reading frame of the gene, these were assumed to be artefacts and the reads discarded.

The aim was to obtain a minimum of 1,200 reads per gene per sample, equating to a 99.3% chance of detecting five or more reads representing an allele present at a frequency of 1%. In some cases, this required data from different sequencing runs to be combined in order to obtain sufficient depth. With the exception of the Tp2 gene, the target of obtaining 1,200 usable reads per gene was exceeded for all genes in each of the Muguga cocktail components; the Tp2 gene yielded approximately 500 reads for each component.

## 3 Results

### 3.1 Micro- and mini-satellite genotyping of the Muguga cocktail stocks

**Fig. 1:**
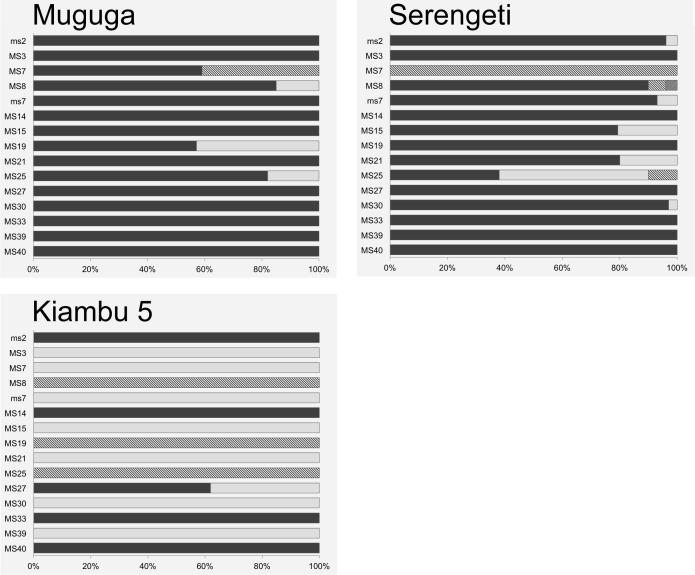
Illustration of the numbers and relative frequencies of micro- and mini-satellite alleles detected in the three components of the Muguga cocktail vaccine (Muguga, Serengeti and Kiambu 5). Alleles are denoted by different fill patterns, the black shaded allele representing the most abundant allele in the Muguga isolate.

**Fig. 2:**
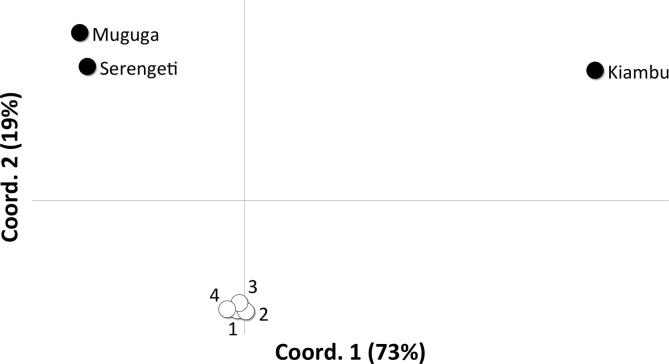
Results of principal co-ordinate analysis of satellite typing data obtained for the individual components of the Muguga cocktail vaccine (Muguga, Serengeti and Kiambu 5) and the four batches of the Muguga cocktail vaccine (ILRI 0801–0804).

Genotypic diversity within the *T. parva* stocks used in the Muguga cocktail was first assessed using a panel of 15 micro- and mini-satellite markers distributed across the four parasite chromosomes. The use of capillary electrophoresis to detect micro- and mini-satellite alleles also allowed estimation of the relative proportions of genotypes present in each parasite sample. The alleles detected and their relative representation within the three parasite stocks are shown in Fig. 1 and the overall diversity of alleles in the Muguga cocktail is summarised in Table 2. Overall, each of the stocks showed limited intra-stock diversity. Only a single allele was detected for 14 of the 15 loci examined in the Kiambu 5 stock; two alleles were found for the remaining locus. Similarly, a single allele was detected with 11 of the satellite markers in the Muguga stock and with eight of the satellite markers in the Serengeti stock. The remaining four satellite markers in the Muguga stock and five of the remaining markers in Serengeti detected a single additional allele, while two additional alleles of MS8 and MS25 were detected in the Serengeti stock. The genotypes of Muguga and Serengeti stocks exhibited a high level of similarity, with at least one allele shared between the stocks for each of the markers. By contrast, the predominant allele detected with 10 of the 15 satellite markers in the Kiambu 5 stock was either unique to this stock (three markers) or present as a minor component (<20%) in either or both of the other stocks. PCoA based on the level of genetic differentiation between each stock illustrates a high level of similarity between the Muguga (Stabilate 4230) and Serengeti (Stabilate 4229) stocks and the distinct profile for the Kiambu 5 stock (Fig. 2).

**Fig. 3:**
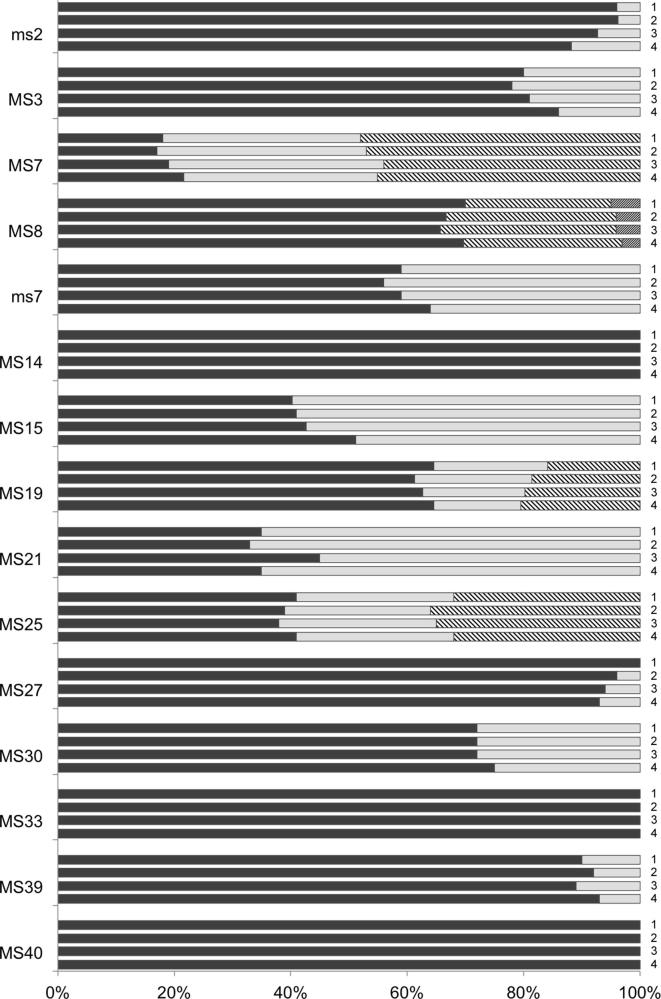
Illustration of the number and relative frequencies of micro- and mini-satellite alleles detected in four batches of the Muguga cocktail vaccine (ILRI 0801–ILRI 0804, labelled 1–4). Alleles are denoted by different fill patterns, employing the same key used in Fig. 1.

Subsequently, micro- and mini-satellite typing was conducted on four batches of the Muguga cocktail vaccine (ILRI 0801–0804) to determine whether the composition of the parasite cocktail reflected that of the individual components described above (Muguga, Serengeti and Kiambu 5). The results are presented in Fig. 3. All alleles found in the individual components were present in the vaccine batches, with the exception of a minor allele of MS8, which was below the threshold value for acceptance. Similarly, all alleles identified in each of the vaccine batches could be identified in one or more component stocks. In addition, there was a high level of consistency between batches, both in terms of the alleles detected and their relative abundance. PCoA revealed a close similarity between the four Muguga cocktail batches, illustrated in Fig. 2 as a tight cluster with little differentiation between batches (*F*_ST_ = 0.003 ± 0.001). Given that the Muguga and Serengeti components were highly similar to one another and that they constitute two-thirds of the vaccine cocktail, it was anticipated that the vaccine batches would be more similar to these components than the Kiambu 5 component and this was confirmed by the PCoA analysis.

### 3.2 Antigen gene sequence diversity in the Muguga cocktail component stocks

**Fig. 4:**
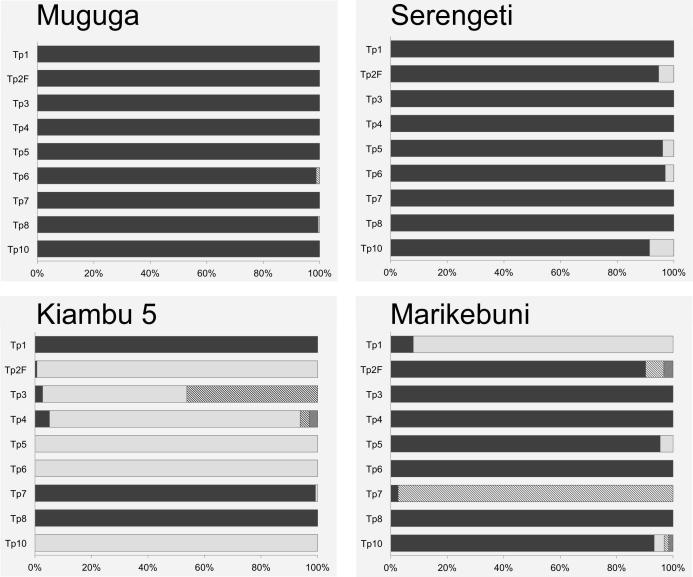
Illustration of the numbers and relative frequencies of antigen gene alleles found using high throughput multi-locus sequencing of genes encoding CD8 antigens in the three components of the Muguga cocktail vaccine (Muguga, Serengeti and Kiambu 5) and the Marikebuni stock. Alleles are denoted by different fill patterns, the black shaded allele representing the most abundant allele in the Muguga isolate. The results for Tp2 (Tp2F) are based on forward sequence reads only. The National Center for Biotechnology Information (NCBI, USA) sequence read archive (SRA) accession number for these sequence data is SRP065583.

Genotypic diversity within and among the *T. parva* stocks that constitute the Muguga cocktail was further investigated by multi-locus sequencing of genes encoding CD8^+^ T cell antigens by applying high throughput sequencing using Roche 454 technology. The number of alleles detected for each gene and their relative proportions in each stock are illustrated in Fig. 4. Details of the alleles identified among component stocks of the Muguga cocktail are summarised in Table 3. With the exception of a single di-nucleotide deletion within an intron region of one Tp10 allele, all allelic differences were due to point mutations.

Despite the considerable sequencing depth achieved, the number of allelic variants identified in each of the component stocks was low (one to four alleles). With the exception of one gene in Kiambu 5 (Tp3), a single dominant allele accounted for more than 90% of the reads obtained for each gene in each of the three stocks. Moreover, the alleles detected within each stock and in the different stocks showed a high level of sequence identity (>95%), with the majority of the nucleotide differences not resulting in amino acid polymorphism (Table 3). Amino acid residue polymorphism was found only among alleles of Tp2, Tp3, Tp7 and Tp10.

In the Muguga stock, only a single allele was detected for each gene except Tp6, which had a second allele present at low abundance (0.77%), containing four synonymous substitutions in the 291 nucleotide sequence with respect to the major allele. Across the loci, the same predominant alleles found in the Muguga stock also dominated the Serengeti stock, accounting for all of the sequence reads obtained for five of the genes and more than 90% of reads for the remaining four genes (Tp2, Tp5, Tp6 and Tp10). An additional allele was detected for the latter four genes, which differed from the reference Muguga allele by between two and 10 nucleotide positions. With the exception of the two mutations in Tp2 (see Table 3), these differences did not result in an alteration to the predicted amino acid sequence. A single allele was detected for six of the genes in the Kiambu 5 stock, two of which (Tp1 and Tp8) were identical to that found in the Muguga stock and four (Tp2, Tp5, Tp6 and Tp10) were identical to the minor alleles found in the Serengeti stock. The remaining three genes, Tp3, Tp4 and Tp7, shared one allele with Muguga or Serengeti stock, but also had between one and three additional alleles. The two additional alleles for Tp3 had residue changes over nine and 10 nucleotide positions compared with the Muguga allele, leading to three and four amino acid changes, respectively. The three variant Tp4 alleles had between 21 and 31 nucleotide substitutions compared with the Muguga allele, but all were within an intron and hence did not alter the coding sequence. The Tp7 variant allele contained seven nucleotide substitutions over the length of the amplicon (253 bp), leading to only one amino acid change.

### 3.3 Antigenic diversity in the Muguga cocktail component stocks

Previous studies have identified CD8 epitopes in six of the nine antigen genes examined (Graham et al., 2008) and a single epitope in Tp10 is reported in the present study. Alleles containing amino acid coding changes were only detected in two of these genes. The single amino acid substitution found in Tp7 lies outside the epitope-encoding region. However, the two amino acid changes in Tp2 both lie within one of the defined epitopes (Tp2_27–37_) and result in substitutions at positions 2 (H→D) and 6 (K→N) of the epitope (Table 4).

### 3.4 Analyses of Tp9 sequence diversity by Sanger sequencing

#### 3.4.1 Demonstration of sequence polymorphism in the Tp9 gene

**Supplementary Fig. S1:**
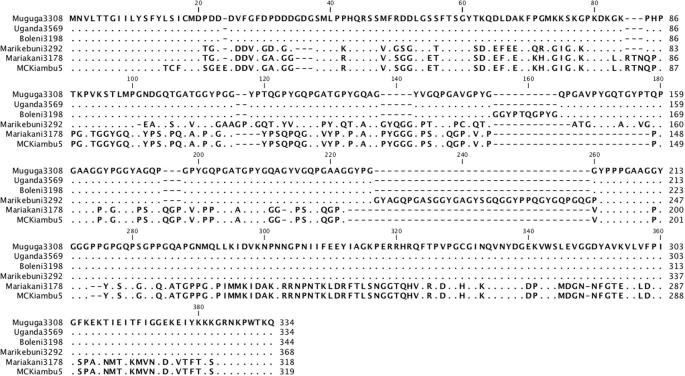
Amino acid sequences of Tp9 alleles obtained from five cloned *Theileria parva* populations (Muguga 3308, Uganda 3569, Boleni 3198, Marikebuni 3292 and Mariakani 3178) and a variant allele (MC Kiambu 5) detected in the Kiambu component of the Muguga cocktail vaccine. The Muguga 3308 sequence, which is identical to the reference genome sequence, is shown as the reference sequence. Amino acid residues conserved in the other alleles are denoted by dots and deleted regions are denoted by dashes.

The initial failure to design primers that could reproducibly generate PCR products suitable for 454 sequencing from the Tp9 gene suggested that this gene is polymorphic. Sanger sequencing was therefore conducted on full-length PCR products from cloned sporozoite stabilates of five cattle-derived *T. parva* isolates – Muguga, Boleni, Uganda, Mariakani and Marikebuni (Morzaria et al., 1995). The Tp9 sequences obtained from the cloned Muguga and Uganda parasites were identical to the 1,005 bp reference Muguga gene, but each of the other clones contained a different allele (GenBank IDs: JN828541, JN828544, JN828549, JN828552). The Boleni sequence was similar to the Muguga reference, differing only by the presence of a 30 nucleotide insert in the central region of the gene. The Mariakani and Marikebuni Tp9 sequences were highly divergent from the reference due to numerous amino acid substitutions as well as several insertions and deletions. The latter resulted in differences in the length of the coding region (957 bp for Mariakani and 1,107 bp for Marikebuni). Translation of these sequences confirmed four alleles of the Tp9 protein (Supplementary Fig. S1). The CD8^+^ T cell epitope identified in Tp9 exhibited amino acid substitutions in six of the nine amino acids in both the Marikebuni and Mariakani sequences, which differed from each other at two residues.

#### 3.4.2 Analysis of Tp9 in the Muguga cocktail components

PCR amplicons of Tp9 obtained from a series of cloned *T. parva*-infected cell lines derived from each of the three Muguga cocktail stocks were subjected to Sanger sequencing. These cloned lines comprised 20 lines infected with Muguga parasites, 10 with Serengeti and 11 with Kiambu 5. Sequences were also determined for Tp1 and Tp2. The findings for both of these genes were consistent with the results of the 454 sequencing, in that only one variant sequence of Tp2, present in all of the Kiambu clones and in all except two of the 10 Serengeti clones, was found. In the case of Tp9, all of the sequences of the Muguga and Serengeti clones were identical to the genome reference sequence, whereas all 11 clones of Kiambu 5 had a distinct allele (Supplementary Fig. S1), which was very similar to that described above for the Mariakani isolate, differing only at five nucleotide positions and encoding an additional amino acid residue within the first 17 nucleotides.

### 3.5 Antigen gene sequence diversity in the Marikebuni stock

Multi-locus 454 sequencing of the same set of nine genes was performed on the Marikebuni stock of *T. parva.* The results are illustrated in Fig. 4 and summarised in Table 5. Single alleles, which were identical to the Muguga genome reference sequence, were detected for four of the genes (Tp3, Tp4, Tp6 and Tp8). Two alleles were identified for three genes (Tp1, Tp5, and Tp7) and four alleles for the remaining two genes (Tp2 and Tp10). Six of these alleles (one for Tp1, two for Tp2, one for Tp7 and two for Tp10) were distinct from those found in the Muguga cocktail. In all cases, a single allele accounted for more than 90% of the sequence reads. With the exception of Tp2, the level of sequence divergence between alleles was very low. The sequence polymorphism resulted in amino acid substitutions in defined CD8^+^ T cell epitopes in only two of the antigens (Table 5). A single allelic variant was found for the Tp1_214-224_ epitope and two allelic variants were detected for all three Tp2 epitopes represented in the partial sequence reads obtained for this gene (Tp2_27–37,_ Tp2_40–48,_ Tp2_49–59_). The epitope variants differed by between one and six amino acid residues from the reference sequence (Table 4).

Sanger sequencing of 17 clones of the partial Tp9 DNA amplified by PCR from the Marikebuni stock revealed four alleles, including one identical to the Muguga reference sequence (11 clones). A further allele differed by only two nucleotides from the reference sequence (two clones), leading to one amino acid change. The other two alleles were similar to each other (two clones each) (only differing at one nucleotide position, leading to an amino acid change), but differed from the Muguga allele at 44 nucleotide residues, leading to 28 amino acid substitutions, and had a 9 bp deletion.

### 3.6 Comparison of diversity with that of field populations of *T. parva*

Previous studies of the sequences of the Tp1 and Tp2 genes in field populations of *T. parva* have shown that both genes exhibit extensive allelic diversity, which was particularly marked in buffalo-derived parasite isolates (Pelle et al., 2011). Herein, we have also detected four alleles of the Tp9 gene in a small sample of cattle-derived parasite isolates. A comparison of these findings, with sequence data obtained for Tp1, Tp2 and Tp9 from the Muguga cocktail and the Marikebuni stock, is presented in Table 6. In contrast to the large number (more than 25) of amino acid sequence variants of Tp1 and Tp2 detected by Pelle et al. (2011) in 82 field isolates of *T. parva*, the Muguga cocktail contained no Tp1 variants and only a single variant allele of Tp2 in addition to the reference Muguga alleles of the two genes. Moreover, the Tp2 variant was antigenically similar to the reference sequence, differing at only one of the six defined CD8^+^ T cell epitopes identified in this antigen. The more limited sequence data obtained for Tp9 revealed only one amino acid variant, in addition to the genome reference sequence, within the Muguga cocktail. Hence, with respect to these antigens, the Muguga cocktail incorporates very little of the observed diversity in field populations of *T. parva.*

The results obtained for the Marikebuni stock revealed one, two and three sequence variants of Tp1, Tp2 and Tp9, respectively, in addition to the genome reference sequence. These included alleles that showed extensive divergence in the encoded amino acid sequence including the CD8^+^ T cell epitopes. Hence, the Marikebuni stock contains greater diversity in these antigens than the Muguga cocktail.

## 4 Discussion

The aim of this study was to use multi-locus sequencing of genes encoding currently identified CD8^+^ T cell antigens in combination with micro- and mini-satellite genotyping, to determine the extent of genetic and antigenic diversity in the components of the Muguga cocktail live *T. parva* vaccine. The results indicate that, overall, the Muguga cocktail vaccine incorporates very little of the genetic and antigenic diversity observed in field populations of *T. parva*.

Roche 454 sequencing of PCR amplicons of antigen-encoding genes was utilised in order to provide greater resolution than had been achieved in previous studies. Preliminary experiments indicated reliable detection of sequence variants present at 0.4% using this method. Use of capillary electrophoresis to detect micro- and mini-satellite alleles also allowed estimation of the relative proportions of genotypes present in each parasite sample. In agreement with previous micro- and mini-satellite genotyping results obtained by Oura et al. (2004), a limited amount of diversity was observed within the components of the Muguga cocktail. Despite the higher resolution afforded by high-throughput sequencing with respect to the detection of rare alleles, this method also revealed limited but appreciable diversity within the vaccine components. A major contributory factor to the overall limited diversity within the Muguga cocktail as a whole was the large number of loci where the Muguga and Serengeti components shared either a single or a clearly predominant allele. This similarity is re-inforced by the whole-genome sequencing effort recently applied to the vaccine component stocks using a gene capture method (Norling et al., 2015), which did not provide evidence of intra-component diversity. However, interestingly, in that study no bi-allelic single nucleotide polymorphisms (SNPs) were identified within the Serengeti transformed stock (which we found to be relatively homogeneous) while 18 SNPs were identified within Kiambu 5. While the number of putative SNPs was noted to be within the error rate of the sequencing platform used in that study and the component stock assumed to be clonal, the present targeted amplicon sequencing approach clearly demonstrates that Kiambu 5 is heterogeneous with, for example, the Tp3 locus displaying three alleles and an estimated heterozygosity of approximately 53% (Fig. 4). The discrepancy is almost certainly due to the greater ability of the methods used in the current study to detect alleles present at low frequency.

In view of the diverse origins of the parasite stocks, it is surprising to find such a high degree of similarity between the Serengeti and Muguga parasites. The Serengeti stock was originally isolated from buffalo (Young and Purnell, 1973), in which the *T. parva* population is known to possess enormous genotypic diversity (Oura et al., 2011, Pelle et al., 2011). Although buffalo *T. parva* parasites readily infect cattle and cause severe disease, they usually differentiate poorly to the tick-infective intra-erythrocytic stage and therefore onward transmission via the tick either does not occur or does so with very low efficiency (Barnett and Brocklesby, 1966, Mbizeni et al., 2013). The limited diversity in the Serengeti stock could be explained by a bottleneck in the cattle-tick cycle, in which only a small subset of the parasites in the original Serengeti could adapt to continued passage through cattle and ticks. However, the likelihood that the selected parasite would be highly similar to the Muguga isolate seems remote and therefore these findings raise the possibility that the Serengeti stock has become contaminated with parasites from the Muguga stock at some time following its isolation from the field. This has also been suggested recently by Norling et al. (2015), based on genome sequence data.

The parasite stocks currently used for the production of the Muguga cocktail have undergone several cattle-tick-cattle passages since the formulation of the Muguga cocktail in the 1970s and, at least for the Serengeti stock, it appears very likely the composition of the stock has changed since its isolation. Micro- and mini-satellite DNA typing and high-throughput multi-locus sequence typing reveal the presence of alleles at a low frequency in the vaccine and each of its component parts. As each component has been maintained in isolation, it is logical to view each as a sub-population of *T. parva* genotypes. Field studies of *T. parva* have previously documented appreciable polymorphism at a number of the antigen (Pelle et al., 2011) and satellite marker loci (Oura et al., 2005, Oura et al., 2011) used in this study. Moreover, mixed genotypes were frequently detected in single samples from naturally infected animals (Oura et al., 2005). It is interesting to note that within each of the three individual vaccine component stocks, the majority of loci display no polymorphism while others show two to four alleles. In broad terms, therefore, the genotypes within a component can be said to share a remarkably high degree of similarity. This lies in contrast to the low level of similarity which two unrelated field isolates would be expected to share. It can therefore be surmised that each component stock represents a highly inbred parasite population. As each stock has undergone extensive cattle-tick-cattle passage in isolation, it is likely that the present day stocks represent an inbred population derived from the original field isolates. Each component is genetically isolated and will have a small effective population size. The parasite may, therefore, be subjected to significant bottlenecking through stochastic processes related to the limited number of cattle/ticks used to maintain the population coupled with the potential for selection of particular genotypes in the cattle host. Consequently, it may be hypothesised that two evolutionary ‘drivers’ will act to reduce the diversity at each locus – selection and genetic drift.

Mathematical modelling of field data suggests that a large proportion of tick transmissions of *T. parva* are from carrier cattle, i.e. animals with persistent infection following resolution of the acute phase of infection (Medley et al., 1993). There is also evidence that carrier cattle can acquire infection with additional parasite genotypes following tick challenge, thus resulting in carriage of mixed genotypes (Oura et al., 2007), which have the potential to recombine during subsequent transmission by ticks. The selective pressures in the field, where carrier infections play a prominent role in transmission, are likely to be different than those experienced by laboratory stocks, which are maintained in isolation by repeated tick passage from acutely infected cattle. Hence, it seems likely that different parasite genotypes will have differing levels of fitness in the course of this regime of passage through the parasite life-cycle. Consequently, genotypes with loci conferring a long-term passage ‘fitness’ would be positively selected in each component population and genetically linked regions surrounding such loci would also be selected by way of a genetic hitch-hiking effect. In addition to any selective pressure, given that the parasite population is relatively small and isolated, it is likely that genetic drift will result in alleles being lost from each component population in a stochastic manner during the course of successive passages. It has been demonstrated previously that changes to the parasite population can occur with additional cattle-tick passages for the Marikebuni stock, a stock that has also been used for immunisation in the field (Katzer et al., 2006). The current study demonstrated that an early passage of the Marikebuni stock (stabilate 3014) contained relatively greater diversity than any of the Muguga cocktail components. However, by applying micro- and mini-satellite genotyping to sequential passages of this stock, Katzer et al. (2006) identified changes in allele frequencies at each passage. This resulted in a stock that was quite distinct from the original after only three rounds of passage, with a minor component becoming dominant in the latter. It is of note that the Muguga stock, which has very limited diversity, has been subjected to prolonged passage, having been maintained by regular tick passage following its isolation in 1961 until the early 1970s when procedures for cryopreservation were developed (Cunningham et al., 1973). Unfortunately, without archive material with which to determine the early composition of the component stocks of the Muguga cocktail, it is impossible to estimate the scale of loss of genetic diversity through time. Nevertheless, the possibility of genetic selection/drift poses a clear and present threat to the stability of each of the vaccine components as they are maintained by cattle-tick passage, with low frequency alleles being at greatest risk. Although the present study demonstrates that four batches of vaccine prepared at different time points from the same batches of infected ticks show very similar composition of parasite genotypes, it is unknown how this consistency is maintained when vaccine batches are produced using stabilates that have undergone additional cattle-tick passage. Another concern is whether the genetic diversity within each component will be present within every dose of the Muguga cocktail vaccine. One of the goals of early studies with cryopreserved *T. parva* sporozoites was to establish a dose of parasites that would result in mild clinical reaction with recovery in all animals, which could be used for vaccination. However, titrated doses of sporozoites resulted in variable reactions; at doses that did not give severe reactions in any animals, some of the animals did not become infected, as judged by the absence of detectable parasites and susceptibility to subsequent parasite challenge (Cunningham et al., 1974). Moreover, there was a relatively narrow dose range (100 fold) between most animals developing severe reactions and some animals not becoming infected, suggesting that a few thousand or fewer infection events in vivo are sufficient to result in severe disease. Hence, at the doses used for infection and treatment, it is uncertain whether low frequency parasite genotypes will be present in every vaccine dose and whether they make a meaningful contribution to the breadth of immunity induced by vaccination.

Despite the concerns regarding parasite composition, vaccination of cattle with the Muguga cocktail appears to be effective in the field (Di Giulio et al., 2008). The obvious question arising from the results of the current study is why, given its limited diversity, is it able to provide broad protection? Our current knowledge of the immunological basis of strain specificity of immunity following immunisation with individual parasite isolates indicates that it is a consequence of strong immunodominance hierarchies in the antigens recognised by the specific CD8^+^ T cell response, resulting in the response in each animal being focused on a few antigens, the most dominant of which tend to be polymorphic in the parasite population (reviewed in Morrison et al., 2015). The antigen dominance hierarchy varies between animals of different MHC genotypes and can be influenced by the parasite isolate used for immunisation (Taracha et al., 1995b). The latter, in part, reflects the capacity of the parasite to undergo frequent sexual recombination during transmission of heterogeneous parasite populations (Oura et al., 2005, Katzer et al., 2011), such that allelic variants of different antigens can occur in different combinations in different parasite genotypes. The specificity of responses induced by immunisation with mixtures of parasite isolates has not been investigated. However, since the antigens expressed by each constituent parasite genotype will infect different cells and present a different but overlapping repertoire of antigens to responding T cells, each parasite might be expected to generate its own CD8^+^ T cell response against different dominant antigens. Consequently, the overall CD8^+^ T cell response is likely to comprise a wider range of antigenic specificities than that induced by the individual parasites, increasing the likelihood that one or more of the specificities will recognise parasite genotypes encountered upon field challenge. In this model (elaborated further in Morrison et al., 2015), the immunising parasites do not necessarily need to include all of alleles of each polymorphic antigen to generate broad protection. If this model is correct, the precise parasite populations used within a live cocktail vaccine may not be critical, providing they are relatively antigenically diverse. To what extent this scenario applies to other protozoal pathogens remains unclear. Among those referred to earlier, immunity to *Eimeria* spp. is mediated predominantly by T cell responses (Smith and Hayday, 2000) and data from cross-immunity studies indicate that a similar immunodominance phenomenon may account for the lack of cross-protection between of isolates of *Eimeria maxima* (Smith et al., 2002).

On the basis of the model proposed above and given the results of the current study, we propose that an improved *T. parva* cocktail could be produced using a mixture of genotypically diverse cloned parasites. Given that the Muguga cocktail essentially consists of two major components, we hypothesise that three genotypically distinct clones would be sufficient to induce a similar or greater breadth of protection than the Muguga cocktail. Methods for obtaining sporozoite preparations from cloned parasites are well established (Morzaria et al., 1995) and genome sequencing could be employed to aid selection of the most genotypically and antigenically diverse clones. Field trials would be required to test the efficacy of such a vaccine. The use of cloned parasites would eliminate the concerns associated with the loss of minor genotypic components from uncloned parasite populations and enable the parasite composition of the vaccine to be measured with greater precision, thereby improving the quality control of vaccine batches.

However, until a more standardised vaccine is formulated, it remains essential to monitor the composition of different batches of the current vaccine, especially if the stocks used for vaccine production have been subjected to additional passages through cattle and ticks. The results of the current study provide useful reference data on which to establish molecular typing methods for monitoring the composition of the vaccine.

## Figures and Tables

**Table 1:** Sequences of *Theileria parva* gene primers incorporated into fusion primers, primer concentrations and annealing temperatures used to generate PCR amplicons from antigen genes for high throughput sequencing.

Gene	Primer sequences (5′–3′)	Concentration (pmol/reaction)	Annealing temp. (°C)	Amplicon size^a^
Tp1	for-CTGGTGTACAATTTGGTGGG	20	55	428
rev-AACTTNMCTTCTTGCGAACC	20

Tp2	for-ATGAAATTGGCCGCCAGATTA	10	49	492
for-GCCAGATTAATHAGYCTTTAC	10
rev-AGATTTGTCACTAYCTGTWBYAGG	25
rev-AGATTCGTCCTCAYCTGTWBYAGG	25

Tp3	for-AGCAGATTTCACTCAAGCTGC	20	54	407
rev-TCCCCCAGAACATTAAACGG	20

Tp4	for-GCAACACAATACTTTGCAGG	10	54	424
rev-CCTCAAACACWCCACAAGTTCC	10

Tp5	for-GTATGCTCGGTAATGGCAG	10	55	347
rev-GATTTTGGTCGCTTCAGGC	10

Tp6	for-CGTCCAATAATTTACGATGTGAG	10		
rev-GCTTAAGTGGGTTAAGGAGACA	10	55	326

Tp7	for-TGAAGAAGGACGACTCGCAC	20	58	292
rev-TCCTCGTCAGTGACGTCGG	20

Tp8	for-ATCCACAACCAAGTGCCCAG	10	54	305
rev-TGCTATTGCGAGTCAACAG	10

Tp10	for-GGTCGTCTGACAATAACC	10	49	314
rev-CTAMCATGTAAATCCAGC	20

a The expected amplicon sizes (excluding the adaptor and Multiplex Identifier (MID) sequence of the fusion primer), based on the Muguga reference genome sequence.

**Table 2:** Summary of numbers and alleles and their lengths (bp) detected in the Muguga cocktail *Theileria parva* vaccine by typing with 15 mini- and microsatellite markers.

^a^Those listed in the first horizontal row are the alleles represented by black fill in Fig. 1, Fig. 3. The alleles in the reference genome are indicated by grey shading.

**Table 3:** Numbers of *Theileria parva* antigen gene sequence alleles and total number of polymorphic residues present in allelic variants detected in the components of the Muguga cocktail vaccine (Kiambu 5, Serengeti and Muguga).

Gene	Total number of sequence reads^a^	Polymorphic nucleotides	No. alleles (nucleotide level)	Polymorphic amino acids	No. alleles (amino acid level)
Tp1	6,156	0/393	1	0/131	1
Tp2	1,596	2/290	2	2/96	2
Tp3^b^	7,196	16/366	3	7/110	3
Tp4^b^	4,314	41/382	4	0/40	1
Tp5^b^	16,214	4/308	2	0/62	1
Tp6	4,263	10/291	3	0/96	1
Tp7	13,356	7/253	2	1/84	2
Tp8	11,364	0/262	1	0/87	1
Tp10^b^	12,360	3/277	2	1/59	2

a The total of sequence reads for the Muguga cocktail is the sum of reads obtained for each of the individual stocks.

b These genes contain introns, accounting for the discrepancies between nucleotide and amino acid lengths.

**Table 4:** Amino acid sequences of variant CD8 T cell epitopes^a^ detected within the Muguga cocktail and the Marikebuli stock of *Theileria parva*.

^a^Residues showing amino acid substitutions compared with the reference sequence are bold and highlighted in grey.

**Table 5:** Numbers of *Theileria parva* antigen gene sequence alleles and total number of polymorphic residues present in allelic variants detected in the Marikebuni stock.

Gene	Total number of sequence reads	Polymorphic nucleotides	No. alleles (nucleotide level)	Polymorphic amino acids	No. alleles (amino acid level)
Tp1	290	4/393	2	2/131	2
Tp2	1,484	107/290	3	53/96	3
Tp3^a^	352	0/366	1	0/110	1
Tp4^a^	530	0/382	1	0/40	1
Tp5^a^	4,771	4/308	2	0/62	1
Tp6	1,933	0/291	1	0/96	1
Tp7	1,875	3/253	2	0/84	1
Tp8	15,445	0/262	1	0/87	1
Tp10^a^	12,267	3/277	4	1/59	2

a These genes contain introns, accounting for the discrepancies between nucleotide and amino acid lengths.

**Table 6:** Comparison of the number of alleles detected for the Tp1, Tp2 and Tp9 gene sequences in the Muguga cocktail vaccine stabilates and the Marikebuni stock, with the number of alleles detected previously in field isolates of *Theileria parva.*

Source of parasite material	Number of isolates	Tp1 alleles		Tp2 alleles		Tp9 alleles	
DNA	Amino acid	DNA	Amino acid	DNA	Amino acid
Vaccine stocks							
Muguga cocktail	3	1	1	2	2	2	2
Marikebuni	1	2	2	3	3	4	4

Field isolates^a^							
Cattle	41	12	10	5	4	nt	nt
Buffalo	41	26	26	38	38	nt	nt

nt, not tested.

a Numbers of alleles detected by sequencing Tp1 and Tp2 genes in 82 field isolates of *T. parva* reported by Pelle et al. (2011).
